# Reinforcing Urea–Formaldehyde Resins with Low-Cost, Mechanically Derived Nanocellulose: A Sustainable Approach

**DOI:** 10.3390/molecules30142911

**Published:** 2025-07-10

**Authors:** Eleni A. Psochia, Emmanouil Karagiannidis, Eleftheria Athanasiadou, Konstantinos S. Triantafyllidis

**Affiliations:** 1 Department of Chemistry, Aristotle University of Thessaloniki, 54214 Thessaloniki, Greece; 2 CHIMAR HELLAS S.A., 15 Km National Road, Thessaloniki–Polygyros, 57001 Thessaloniki, Greece; manos.karag@ari.gr (E.K.); eathan@ari.gr (E.A.); 3 Chemistry Department, King Fahd University of Petroleum and Minerals, Dhahran 31261, Saudi Arabia; 4 Interdisciplinary Research Center for Refining and Advanced Chemicals, King Fahd University of Petroleum & Minerals, Dhahran 31261, Saudi Arabia

**Keywords:** nanocellulose, aqueous colloidal suspensions, ultrasonication, urea-formaldehyde resins, formaldehyde emissions, particle boards, low-cost, biobased additives

## Abstract

In this work, we present the fabrication of low-cost, stable nanocellulose colloidal suspensions with an average particle size of approximately 160 nm, produced via a straightforward, solvent-free ultrasonication process that eliminates the need for corrosive chemicals or energy-intensive mechanical treatments. The resulting nanocellulose suspensions were utilized as reinforcing additives in urea-formaldehyde (UF) resins, which were subsequently applied in the production of particle boards. This approach addresses the increasing EU regulatory constraints regarding low formaldehyde-to-urea (F/U) molar ratios and the broader need for biobased, eco-friendly alternatives in the wood adhesive industry. Mechanical testing of the nanocellulose reinforced boards revealed notable improvements in the internal bond strength and modulus of rupture, along with a significant decrease in formaldehyde release compared to boards produced with conventional UF resins. These findings highlight the potential of ultrasonication-derived nanocellulose as an environmentally friendly, cost-effective additive to enhance the mechanical performance and reduce the environmental impact of UF-based wood composites.

## 1. Introduction

Urea-formaldehyde (UF) resins maintain a key role in the global industry and are used in a wide range of applications, such as adhesives, abrasives, textiles, foams, or molded compounds [1]. Owing to their exceptional properties, such as good adhesion, high reactivity, a lack of color, good thermomechanical behavior, and low-cost production, they appear to be ideal candidates for wood adhesive applications [2]. However, due to regulations regarding reduced formaldehyde emissions (FE), UF resins are restricted to a low formaldehyde-to-urea (F/U) molar ratio, resulting in thermosets with poor adhesion and thermomechanical performance due to changes in the resins’ physicochemical characteristics [3,4,5,6]. Therefore, they are usually combined with various fillers, such as nanoclays, montmorillonite, carbon nanotubes, or other carbon-based materials, for the enhancement of their mechanical and adhesion features [7].

However, increasing environmental awareness and the depletion of fossil-based materials have strengthened the drive for renewable and biobased products [8,9,10]. From this perspective, natural fibers derived from various polysaccharides are used for polymer reinforcement owing to their biodegradable and renewable nature [11,12,13,14]. Among them, cellulose nanofibrils (CNFs) are distinguished for their promising mechanical and thermal features [15,16] and are widely applied in various industrial polymer-related fields, such as adhesives, biomedicine, and packaging [17,18].

In this context, various forms of nanocellulose (micro- or nanofibers, nanocrystals) have been used for the reinforcement of UF resins. Numerous studies have reported that the addition of cellulose nanofibrils resulted in improved mechanical features of both the adhesives and the particle boards. Veigel et al. showed improved toughness and bonding performance in wood adhesives after microfibrillated cellulose (MFC) addition [19], while Mahrdt and his coworkers demonstrated improved mechanical board performance [20]. Karagiannidis et al. studied the effects of the stage of the introduction of MFC into the UF system [21], while another study showed that increasing CNF addition by a certain amount resulted in better mechanical properties [22]. A more recent work studied the possible interactions between UF and pure and lignin-containing CNFs, showing that the latter result in improved final particle board properties due to a better interface with the UF substrate [23].

Although many studies have focused on the effects of the addition protocol of CNFs in UF resins, i.e., the addition level or stage of addition, as previously mentioned, limited studies have focused on the type of cellulose nano-/microparticle. Most studies refer to using CNFs deriving from supermasscolloider treatment, while a few have explored cellulose nanocrystals (CNCs) as a potential additive [24,25]. These methods usually require long and/or expensive procedures and equipment or the utilization of organic solvents, reducing their industrial feasibility [26]. To this end, we explore the mechanical production of cellulose nanoparticles, in the form of stable colloidal suspensions, using an ultrasonication probe. While ultrasonication is widely used to produce nanocellulose, to our knowledge, all studies have used it in combination with other treatments, such as grinding, homogenization, or acid hydrolysis [27,28].

In this work, three different concentrations of aqueous suspensions of colloidal nanocellulose were produced via a facile and low-cost ultrasonication approach. Namely, suspensions of 1, 2, and 3% *w*/*v* were produced and incorporated into UF resins, which were subsequently utilized to produce particle boards. The effects of the nanocellulose suspension’s concentration on the resins’ physicochemical characteristics, as well as on the boards’ properties, were evaluated. The stage of cellulose addition into the resins, either during the resin’s synthesis (Approach 1) or as an additive mixed with the already prepared resin in a glue mixture (Approach 2), was also examined. In a final step, the nanocomposite resins were used for the fabrication of particle boards, and their mechanical performance was evaluated.

## 2. Results and Discussion

### 2.1. Nanocellulose Characterization

#### 2.1.1. FT-IR Spectroscopy

**Figure 1:**
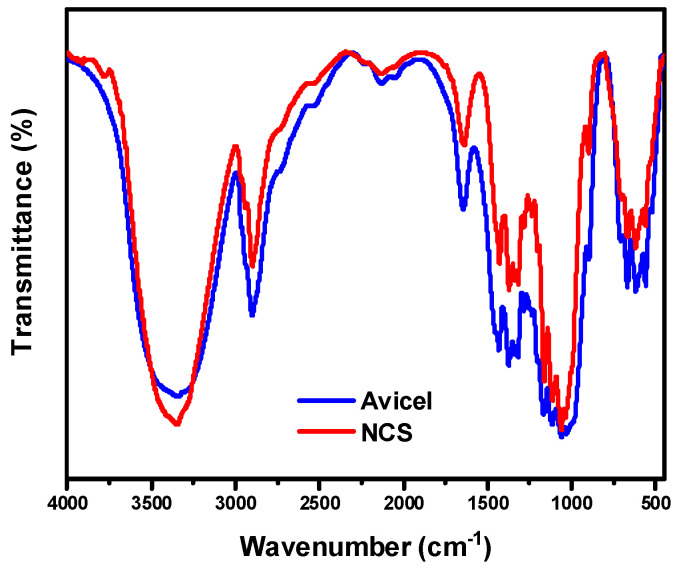
FT-IR spectra of initial Avicel microcrystalline cellulose and NCS nanoparticles.

To evaluate the chemical structure of the produced cellulose nanoparticles after ultrasonication treatment (NCS), FT-IR spectroscopy was employed. As can be seen in Figure 1, all cellulose characteristic bands are present. The prominent band at 3300 cm^−1^ is attributed to free-OH groups’ stretching vibrations, while the bands at 2906 and 1373 cm^−1^ correspond to stretching and deformation vibrations of C-H groups in glucose units. The β-glycosidic linkage absorption at 891 cm^−1^ is also present, suggesting that the mechanical treatment did not affect cellulose’s chemical structure [29].

#### 2.1.2. Particle Size Measurements and Colloidal Stability

**Figure 2:**
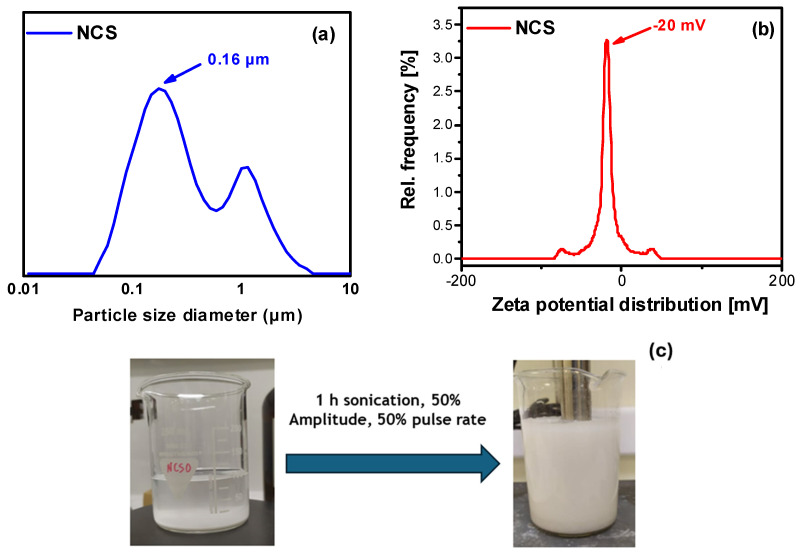
(**a**) Particle size distribution, (**b**) zeta potential measurements of nanocellulose (NCS) particles, and (**c**) photos of aqueous suspension of parent microcrystalline cellulose Avicel (**left**) and of the isolated stable nanocellulose colloidal suspension (**right**).

The particle size of the produced nanocellulose (in the form of stable colloidal suspensions) was determined with DLS measurements (Figure 2). As can be seen in Figure 2a, the NCS particle size ranges from around 100 nm to 1 μm, with a mean particle size diameter of 160 nm. Zeta potential measurements confirmed the formation of stable colloidal cellulosic suspensions with a mean value of 20 mV (Figure 2b). Figure 2c shows the initial aqueous Avicel suspension (5% *w*/*v*) before sonication treatment and the obtained final nanocellulose colloidal (1% *w*/*v*) after 1 hr of ultrasonication and separation/recovery via centrifugation. It can be clearly seen that the parent microcrystalline cellulose (with an average size of 65 μm) sediments at the bottom of the beaker, while the colloidal suspension remains stable for a long period of time (several months).

#### 2.1.3. Cellulose Crystallinity

**Figure 3:**
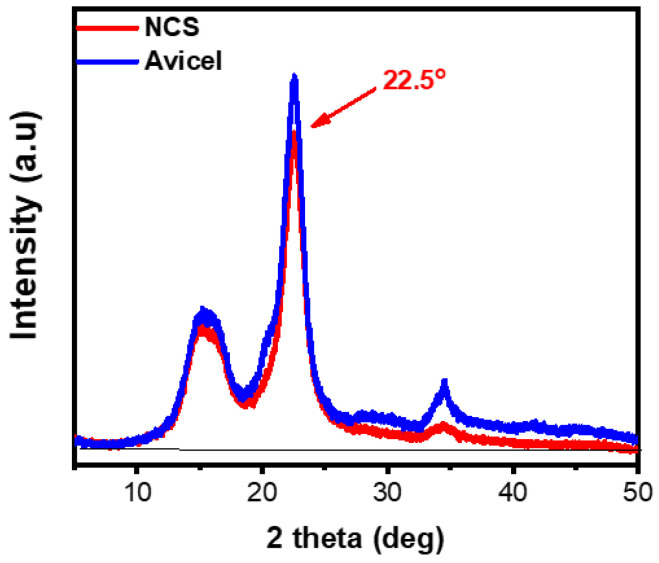
XRD diffractograms of pristine Avicel microcrystalline cellulose and ultrasonication-treated NCS nanoparticles.

X-ray diffraction analysis was performed to evaluate the crystallinity of the produced nanocellulose. Figure 3 shows the obtained diffractograms for pristine Avicel and the isolated cellulose nanoparticles. The produced cellulose nanoparticles are highly crystalline and exhibit all signatures characteristic of the cellulose I structure, namely at 15.2°, 22.5°, and 34.5° corresponding to the (101), (002), and (040) lattice planes, respectively [30]. The XRD analysis revealed that the sonication treatment did not have a significant effect on the cellulose crystalline structure as it resulted in highly crystalline cellulose nanoparticles (NCS). This was confirmed by the very similar crystallinity indices of the initial microcrystalline cellulose (Avicel) and the ultrasonicated NCS micro-/nanoparticles, corresponding to 87% and 86%, respectively.

#### 2.1.4. Morphologies of Parent and Ultrasonicated Cellulose Micro-/Nanoparticles

**Figure 4:**
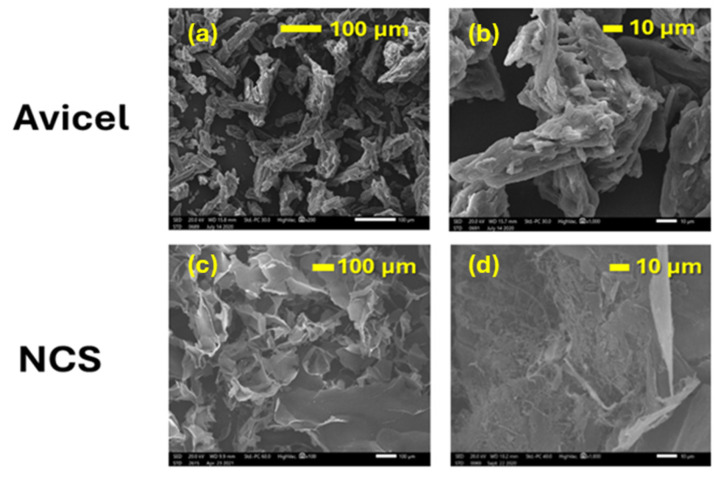
SEM images of (**a**,**b**) initial Avicel cellulose, and (**c**,**d**) freeze-dried colloidal NCS nanocellulose.

The morphologies of the ultrasonicated cellulose particles (NCS) and of the parent Avicel cellulose were studied using scanning electron microscopy (SEM). The respective SEM images are shown in Figure 4. As can be seen, Avicel exhibits rod-like aggregated particles with a width of about 10–30 μm and length of about 65 μm. Following ultrasonication treatment (NCS), the resulting particles consisted predominantly of nanofibers with diameters below 250 nm and lengths exceeding 5 μm, displaying a relatively uniform distribution. Notably, the nanofibers could be identified at the “defects” of larger microsheets (flakes) that were formed by the aggregation of the primary fibers during the freeze-drying of the colloidal nanocellulose solution. These microsheets, characteristic of the lyophilized NCS powders, possessed smooth surfaces exceeding 100 × 100 μm in area and a thickness of roughly 5 μm.

### 2.2. Nanocomposite UF Resin Characterization

**Figure 5:**
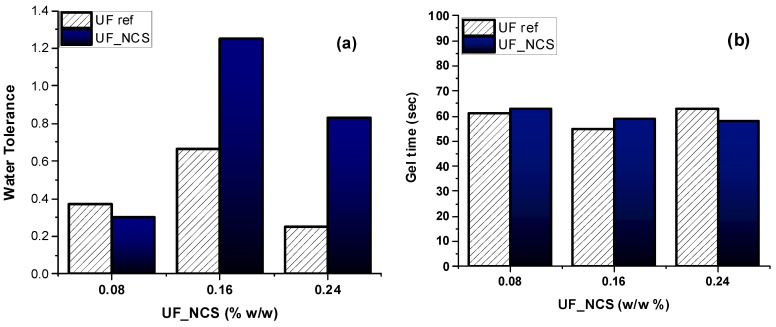
(**a**) Water tolerance and (**b**) gel time properties of UF resins before and after nanocellulose addition in different concentrations.

All nanocellulose-reinforced UF resins resulting from Approach 1 were characterized with typical laboratory analysis to evaluate possible physicochemical changes following cellulose addition. Approach 1 involved the addition of nanocellulose suspensions during the resin synthesis. Therefore, the addition of NCS suspensions with concentrations of 1, 2, and 3% *w*/*v* resulted in UF_NCS nanocomposite resins with 0.08, 0.16, and 0.24 wt.% cellulose (dry basis), respectively. The analysis results are presented in detail in Figure S1 (Supplementary Materials). A notable difference was observed in the resins’ water tolerance after nanocellulose addition at higher levels. It was noted that the resins’ reactivity (gel time) was not significantly different compared to the reference sample, but it was slightly improved when increasing the addition of nanocellulose to 0.24 wt.% (Figure 5). In general, the values indicate no significant differences between the UF reference (UF ref) and the nanocellulose-reinforced resins (UF_NCS).

**Figure 6:**
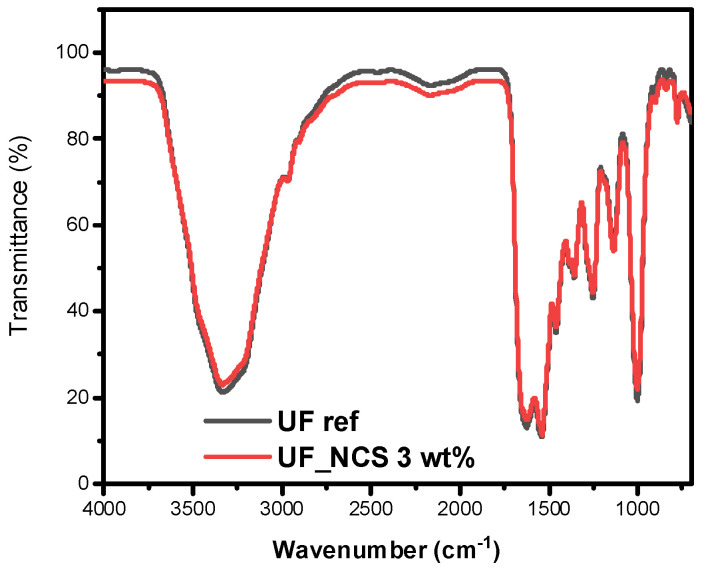
ATR spectra of UF reference and UF_NCS resin.

To further examine possible chemical interactions between the nanocellulose and UF reference resin, the sample prepared with the more condensed nanocellulose colloidal suspension (UF-NCS 3 wt.%)—thus containing the highest cellulose concentration, i.e., 0.24 wt.% dry basis—was examined with ATR spectroscopy. As can be seen in Figure 6, the UF_NCS spectrum is almost identical to that of the UF reference resin, without the emergence of any new peaks, indicating no changes in the chemical structure or the creation of new chemical bonds [31].

**Figure 7:**
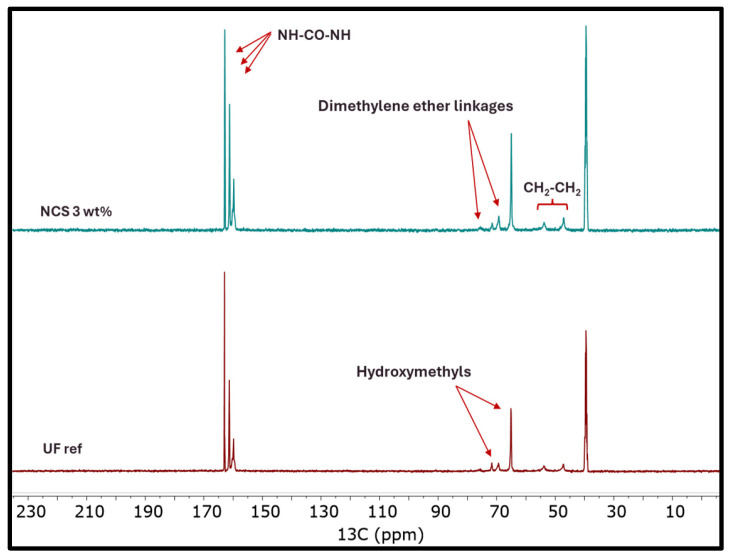
^13^C-NMR spectra of UF reference and UF_NCS resin.

To further investigate any possible chemical interactions or the creation of new bonds after nanocellulose addition, the chemical structure of the nanocomposite resins was also investigated using nuclear magnetic resonance (^13^C-NMR) spectroscopy. From the ^13^C-NMR spectra (Figure 7), the presence of all characteristic groups and bonds of UF resins was confirmed, without the appearance of new features that could be attributed to interactions induced by (nano)cellulose. Thus, it can be suggested that the addition of nanocellulose, at least at the levels studied in this work, does not hinder the development of the urea–formaldehyde polymer network.

**Figure 8:**
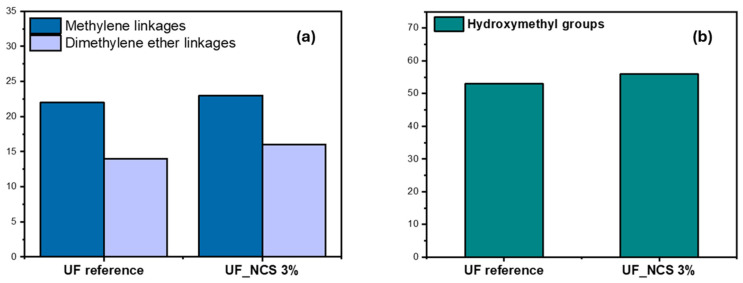
Quantitative ^13^C NMR analysis of UF reference and UF_NCS resins: (**a**) number of linkages and (**b**) hydroxymethyl groups.

Furthermore, a quantitative analysis of the NMR data revealed no significant differences between the nanocomposite resins and the reference resin, except for a slight increase in the proportion of hydroxymethyl groups in the nanocomposite resins, as well as a slightly increased number of methylene and dimethylene linkages (Figure 7 and Figure 8). The increased number of hydroxymethyl groups in the presence of nanocellulose could be attributed to the reactivity of nanocellulose’s surface -OH groups, which may act as sites for formaldehyde activation towards the formation of cellulose-O-CH_2_OH surface groups, thus contributing to the overall hydroxymethyl content of the oligomerized resin [32,33]. As a result, the formation of mono- and bis-hydroxymethylureas is also promoted, both being the first intermediate species of the overall addition/condensation reaction mechanism that takes place.

The observed higher dimethylene ether linkage content after nanocellulose addition is associated with the increased hydroxymehtyl group content. Due to the latter, the condensation reaction between hydroxymethylureas is promoted, thus forming ether linkages (CH_2_-O-CH_2_). Moreover, the hydroxymethyl groups of the UF resin can react with cellulose surface -OH groups, forming cellulose-O-CH_2_-NH- bonds [33]. Finally, nanocellulose’s presence induced also a slight increment in the methylene linkage content. The latter form between the amine groups on urea or hydroxymethylurea molecules. In the presence of nanocellulose, the alignment and local concentration of UF resin chains can be enhanced, owing to the -H bonding formation of cellulose surface -OH groups and UF’s -NH_2_ or -OH groups. This brings the reactive amino groups closer, resulting in increased methylene bridge formation [34].

**Figure 9:**
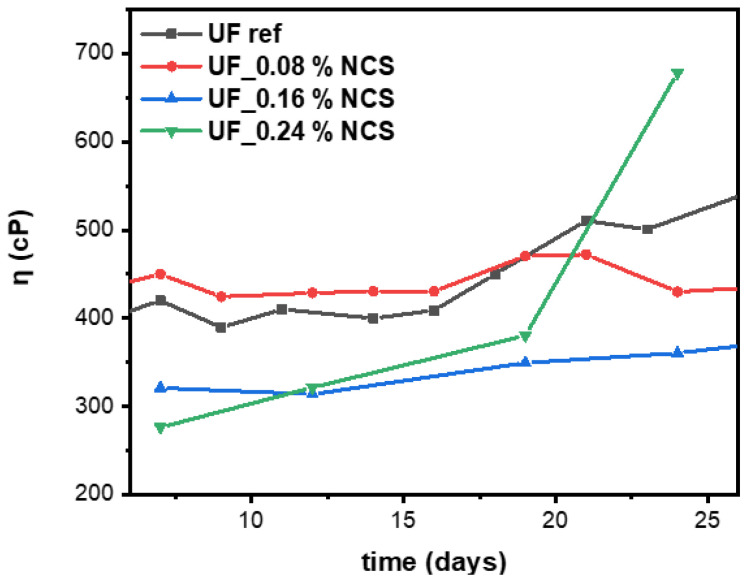
Viscosity values of the UF nanocomposite resins at various time intervals. UF reference viscosity is also presented for comparative reasons.

Resin stability was evaluated with viscosity measurements at various time intervals, namely at 7, 12, 19, 24, and 30 days. As can be observed in Figure 9, the presence of nanocellulose in lower concentrations improved the resin’s stability. The data analysis showed stable resin viscosity for 17 days for both the reference and the lower-cellulose-concentration nanocomposite resins, i.e., the 0.08 and 0.16 wt.% cellulose composites. The UF reference increased in viscosity from 400 to 500 cp from day 17 to 20, where it remained stable until day 24. During the same period, the same UF nanocellulose-reinforced resins increased in viscosity by around 40 cp, resulting in resins with significantly increased storage stability compared to the UF reference. During the period of 20 to 24 days, the composites’ viscosity remained stable, in contrast to the pristine sample, which showed an increasing trend. Interestingly, the 0.24 wt.% cellulose nanocomposite demonstrated a dramatic viscosity increment of around 300 cp, probably attributed to the developed interactions among the highly concentrated nanocellulose and the UF network. Therefore, the addition of nanocellulose up to 0.16 wt.% improved the resin stability, while higher addition levels significantly increased the resin viscosity with time.

### 2.3. Particle Boards’ Properties

**Figure 10:**
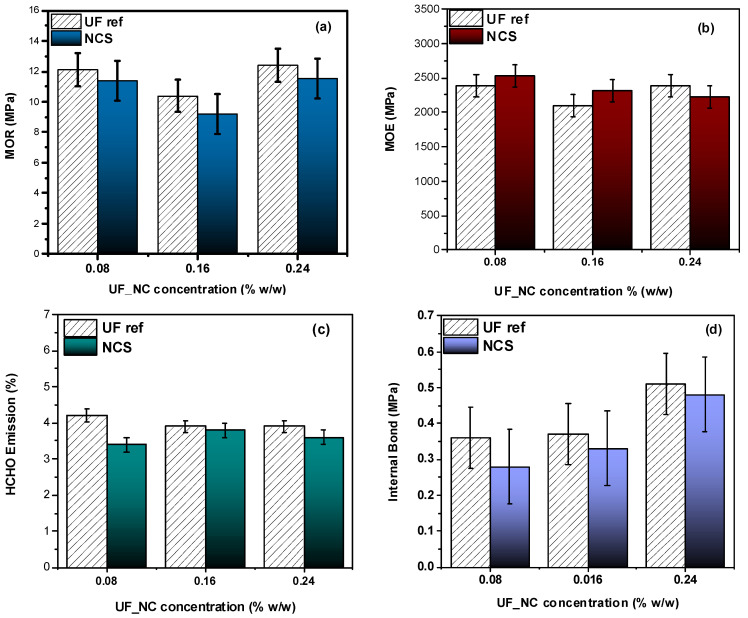
Mechanical properties (**a**,**b**,**d**) and free formaldehyde emissions (**c**) of particle boards produced with UF reference and UF_NCS resins synthesized with Approach 1. The error bars correspond to the standard deviation.

The nanocellulose-reinforced resins were subsequently used to produce particle boards with enhanced performance. NCS addition had a notable impact on the boards’ modulus of rupture (MOR) and modulus of elasticity (MOE). As can be seen in Figure 10a, particle boards prepared with NCS-reinforced UF resins exhibit slightly lower MOR values compared to the pristine ones and therefore more brittle behavior. On the other hand, the nanocomposites show improved MOEs at up to a 0.16 wt.% NCS concentration, while increasing the nanocellulose addition level at 0.24 wt.% leads to a slight MOE decrease (Figure 10b). This decrease could be attributed to the self-agglomeration of cellulose nanoparticles at higher concentrations [33]. Interestingly, all boards with UF_NCS resins exhibited lower formaldehyde (HCHO) emissions (Figure 10c), with the lowest being noticed at lower nanocellulose addition levels. Owing to their increased surface area, the nanoparticles probably absorb free formaldehyde, acting as barriers and preventing its release to the environment. The internal bond (IB) values were slightly lower compared to the pristine resin, which could be associated with inhomogeneous particle dispersion throughout the polymer matrix (Figure 10d).

Cross-sections of the particle boards were also examined by SEM. From the representative images in Figure S2 (Supplementary Materials), no significant differences could be identified in the morphologies of the boards prepared with the use of the UF reference resin and of the nanocellulose-reinforced UF_NCS resin. Furthermore, as expected, no distinct nanocellulose fibers or particles could be distinguished, due to the very low nanocellulose loading levels in the adhesive resin (≤0.24 wt.% on a dry resin basis), considering also that the UF resin (or the nanocomposite UF resin) itself constituted ~10 wt.% of the final board, resulting in effective nanocellulose content in the particle board of <0.03 wt.%.

The thermal properties of the particle boards were studied by DSC via a consecutive heating–cooling–heating protocol (see Materials and Methods), and the respective graphs are shown in Figure S3 (Supplementary Materials). Again, no significant differences were observed between the boards with the reference and nanocomposite UF resins, except the slightly higher glass transition temperature (Tg) in the boards formed with the latter resin. More specifically, the board containing the UF reference resin exhibited a Tg of around 71.5 °C, while the nanocomposite resin-containing board showed a Tg shift to slightly a higher temperature, approximately 76.5 °C. This indicates that the presence of nanocellulose hindered the UF polymer’s chain mobility due to the hydrogen bond formation between its surface hydroxyl groups and the resins’ hydroxyl or amine groups, as also discussed above on the basis of the ^13^C-NMR analysis.

### 2.4. Effects of Nanocellulose Addition Protocol on Particle Boards’ Properties

**Figure 11:**
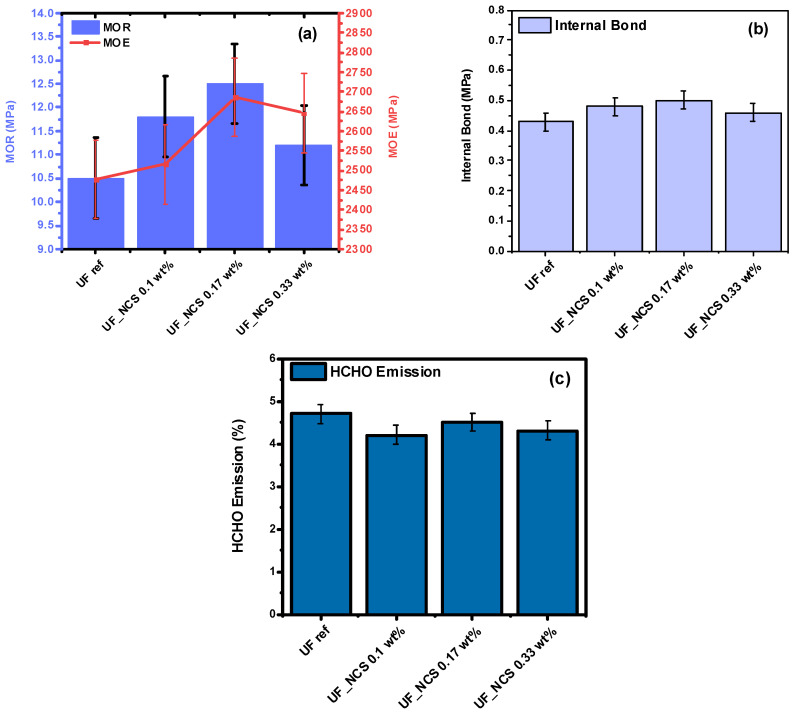
Mechanical properties (**a**,**b**) and free formaldehyde emissions (**c**) of particle boards produced with UF reference and UF_NCS resin prepared by Approach 2. Error bars correspond to the standard deviation.

Similar mechanical tests were performed for the particle boards that were produced using resins that were synthesized with Approach 2 (see Materials and Methods). In Figure 11, the boards’ mechanical properties are presented. As can be seen, the boards with nanocomposite resins exhibit significantly improved MOR and MOE values. Increasing the nanocellulose addition level to 0.17 wt.% on a dry cellulose basis in the liquid resin increased the MOR and MOE values. When the addition level was set to 0.33 wt.%, the boards’ mechanical performance was significantly reduced, but it was still higher compared to the use of the UF reference. The internal bond values were also slightly improved, with the highest values noted for boards prepared with the 0.17 wt.% cellulose nanocomposite resin. The lower board performance for higher nanocellulose loadings in the resin could be associated with cellulose particle agglomeration, owing to cellulose’s hydrophilic nature and tendency to self-aggregate. Finally, boards prepared with nanocellulose-reinforced resins exhibited lower HCHO emissions compared to those with the UF reference. This phenomenon could be attributed to the adsorption of HCHO onto cellulose nanoparticles, inhibiting their release out of the system.

The in situ addition of nanocellulose (Approach 1) did not favor the development of a homogenously dispersed polymer network and therefore the effective reinforcement of the boards’ performance. As shown by the ^13^C-NMR analysis, this is probably attributed to nanocellulose’s interactions with either the hydroxymethyl groups of the UF resin or the formaldehyde molecules, possibly disturbing the crosslinking UF resin, which was subsequently reflected in the boards’ mechanical performance. In contrast to Approach 1, incorporating NCS into the prepared glue mixture (Approach 2) resulted in boards with significantly improved mechanical performance.

## 3. Materials and Methods

### 3.1. Materials

Avicel microcrystalline cellulose (average particle size ~65 μm) was purchased from Sigma-Aldrich (Vienna, Austria) and was used to produce the nanocellulose samples. Formaldehyde 37 wt.% aqueous solution was provided by PanReac (Barcelona, Spain), while urea and sodium hydroxide (NaOH) were provided by the Elton Group S.A. (Thessaloniki, Greece). Ammonium sulfate was supplied by New Trade Fertilizers (Athens, Greece) and was used as a hardener for the UF resins’ production. All resins were synthesized at the laboratory of CHIMAR (Thessaloniki, Greece). All particle boards were manufactured at the pilot plant of CHIMAR using a pilot-scale hydraulic press.

### 3.2. Nanocellulose Production and Characterization

#### 3.2.1. High-Intensity Ultrasonication

The colloidal nanocellulose suspensions were produced via ultrasonication. Namely, aqueous suspensions of microcrystalline cellulose (5% *w*/*v*) were sonicated with an Ultrasonic Vibracell 250 W, at 25 Hz for 1 h, at a 50% amplitude and a pulse rate of 1 pulse/sec. Following a centrifugation step at 3500 rpm for 10 min, the supernatant of the nanocellulose suspension (NCS) was separated from the solid residue (NCS solid), which was collected and dried at 40 °C for 3 days. Finally, nanocellulose aqueous suspensions with concentrations of 1, 2, and 3% *w*/*v* were produced via controlled water evaporation and were later used for the preparation of cellulose-reinforced adhesive mixtures.

#### 3.2.2. Dynamic Laser Scattering (DLS)

The particle sizes of the produced cellulose nanoparticles (in the form of colloidal suspensions) were determined by means of DLS with a Litesizer 500 particle analyzer (Anton Paar, Austria). Prior to analysis, the samples were diluted in water at 1/100 *v*/*v* and were sonicated for 5 min. All measurements were performed in triplicate.

#### 3.2.3. X-Ray Diffraction (XRD)

The crystallinity of the synthesized (dried) cellulose nanoparticles was studied with XRD analysis over the 5^°^–50^°^ 2θ range using a MiniFlex II diffractometer from Rigaku Co. (Rigaku Company, Tokyo, Japan) with the Bragg–Brentano geometry (θ,2θ) and Ni-filtered CuKa radiation (λ = 0.154 nm). The crystallinity index of the cellulose particles was calculated using the Equation Crl (%) = 100 × (I_002_ − I_am_)/I_002_, where I_002_ is the intensity of the (002) peak at about 2θ ≈ 22.5° and I_am_ the intensity of the background at 2θ ≈ 18.3°.

#### 3.2.4. Fourier Transform Infrared Spectroscopy (FT-IR)

The FT-IR spectra of the dried cellulose nanoparticles were obtained using a PerkinElmer FT-IR spectrometer (PerkinElmer, Waltham, MA, USA), model SPECTRUM 1000 (Spectrum 1). The spectra were obtained using KBr pellets and 32 co-added scans between 450 and 4000 cm^−1^ at a resolution of 4 cm^−1^.

#### 3.2.5. Scanning Electron Microscopy (SEM)

The morphological characteristics of the cellulose micro-/nanoparticles were examined via scanning electron microscopy (SEM) on a Jeol (JSM-5600) microscope. Prior to imaging, the nanocellulose colloidal suspensions were freeze-dried and coated with a thin layer of gold to become conductive. SEM observations were conducted at an accelerating voltage of 20 kV.

### 3.3. Synthesis and Characterization of Neat and Nanocomposite UF Resins

#### 3.3.1. Synthesis of Reference and Nanocomposite UF Resins

Urea-formaldehyde resins were synthesized via a two-stage process—methylolation and condensation reactions—following a previously reported method [21,35]. Namely, the methylolation step was marked by the addition of formaldehyde to urea to produce methylolurea entities. The system was stabilized in alkaline conditions with the addition of sodium hydroxide. The methylolation step was followed by condensation reactions at elevated temperatures and in an acidic environment, which was achieved with the use of formic acid. When the required viscosity was achieved, the reaction was ceased, and the pH was adjusted to the alkaline region. Finally, with an additional portion of urea, the final molar F/U ratio was set at 1.0–1.1.

For the preparation of the NCS-reinforced resins, a similar synthesis procedure was followed, and two different addition approaches were studied. The first approach (Approach 1) involved the addition of the nanocellulose colloidal suspension during resin synthesis, while the second (Approach 2) focused on the addition of the nanocellulose to the prepared resin in the glue mixture. Approach 1 involved the addition of NCS suspensions of 1, 2, and 3% *w*/*v* and resulted in the preparation of UF–cellulose resins of 0.08, 0.16, and 0.24 wt.% dry cellulose in the liquid resin, respectively (liquid resin consisted of a colloidal suspension of resin in water with a concentration of 66 wt.% resin solids). During Approach 2, an NCS suspension of 1% *w*/*v* was added to the glue mixture to achieve concentrations of 1, 2, and 3 wt.% of the total resin, corresponding to 0.1, 0.17, and 0.33 wt.% dry cellulose in the liquid resin, respectively.

#### 3.3.2. Physicochemical Characterization of the Liquid UF Resins

All resins were studied following standard lab analysis methods [21,35]. Namely, they were characterized as to their viscosity, gelation time, and water tolerance. The viscosity was assessed using a Brookfield viscometer at 25 °C, using a SC4-13R sample chamber and an SC4-18 spindle (Middleborough, MA, USA). The gelation time was defined by measuring the time needed for resin gelation in boiling water after the addition of 3.5% *w*/*w* (dry/dry resin) ammonium sulfate, which was used as a hardener. The water tolerance of the synthesized resins was evaluated by adding 10 mL of the resin in a volumetric cylinder and determining the amount of water needed until the coagulation of the sample.

The liquid UF resins were also analyzed by attenuated total reflectance (ATR) and ^13^C-NMR spectroscopy (Agilent Technologies, Santa Clara, CA, USA). The ATR spectra of the liquid UF resin samples were acquired using a Cary 670 spectrometer (Agilent Technologies, Palo Alto, CA, USA) equipped with a diamond-attenuated total reflectance (ATR) accessory (GladiATR, Pike Technologies, Madison, WI, USA). Infrared absorbance spectra were recorded over a wavenumber range of 4000–450 cm^−1^, with a resolution of 4 cm^−1^ and 32 co-added scans. The resulting spectra were subsequently baseline-corrected and normalized for analysis.

The various groups and structures in the produced neat and nanocomposite urea–formaldehyde (UF) resins were studied using ^13^C-NMR. In addition, a semi-quantitative analysis based on the same spectra was also performed to quantify the structures/groups of the UF resins. The NMR spectra were obtained on an Agilent 500 MHz spectrometer using deuterated DMSO-d6. In a vial, 800 μL of UF resin was dissolved in DMSO-d6 and left to stir until fully dissolved. The spectra were recorded using 400 scans and a relaxation delay of 8 s. Baseline and manual phase correction was applied, and the NMR spectra were processed with MestReNova (Version 14.0.2-26256, Mestrelab Research).

### 3.4. Particle Board (PB) Production and Testing

#### 3.4.1. Mechanical Testing

All boards were manufactured at CHIMAR (Thessaloniki, Greece), following the typical industrial practice [21]. The boards’ mechanical properties were examined following the European standards (EN) in force. Therefore, measurements were performed to determine features like the density (EN323), tensile strength/internal bonds (IBs) (EN310), bending strength/modulus of rupture (MOR)/modulus of elasticity (MOE) (EN319), and formaldehyde content (perforator method, ISO 12460-5) [21,35].

#### 3.4.2. Particle Board Morphology

Cross-section images of the particle boards produced with the UF reference resin and nanocomposite UF_NCS resins were obtained via scanning electron microscopy analysis (SEM) using a Jeol (JSM-5600) microscope (Tokyo, Japan). Prior to imaging, the samples were sputter- coated with gold to become conductive using a voltage of 20 kV.

#### 3.4.3. Differential Scanning Calorimetry (DSC)

DSC measurements were obtained using a PerkinElmer Pyris Diamond differential scanning calorimeter (Solingen, Germany), calibrated with pure indium, zinc, and tin standards, combined with a cooling system—the PerkinElmer Intracooler 2 (Solingen, Germany). An amount of 5.0 ± 0.1 mg of the sample was sealed in an aluminum pan for the analysis. The first heating scan was conducted at 10 °C/min from room temperature to 260 °C, followed by a cooling step at 10 °C/min to room temperature and a subsequent heating step (2nd heating) to 260 °C at 10 °C/min, to calculate the glass transition temperature (T_g_) after erasing the thermal history of the samples during the 1st heating scan.

## 4. Conclusions

The purpose of this work was to synthesize UF resins using low-cost fabricated nanocellulose as a reinforcing additive following two different incorporation methods. Cellulose nanoparticles in the form of stable aqueous colloidal suspensions were successfully fabricated via ultrasonication. The average size of the colloidal nanoparticles was about 160 nm (DLS measurements). Upon the freeze-drying of the colloidal suspensions, microsized particles were recovered with a flake-like morphology, consisting, however, of small cellulose nanofibers with diameters smaller than approximately 250 nm and lengths of several micrometers (>5 μm). The nanocellulose colloidal suspensions were subsequently incorporated into UF resins at various addition levels. Cellulose addition was achieved either by the in situ preparation of the nanocomposite resin (Approach 1) or at the final stage in the glue mixture (Approach 2). The nanocomposite resins were subsequently used to produce particle boards, aiming at enhanced performance properties. The in situ addition of nanocellulose at higher concentrations slightly improved some of the resins’ and boards’ properties. Between the two methods, Approach 2 was more effective for the enhancement of the boards’ mechanical properties. Increasing nanocellulose addition to up to 0.17 wt.% (dry cellulose in liquid resin) improved the boards’ performance regarding the mechanical properties. In contrast, higher loading levels (0.24–0.33 wt.%) showed a slight decrement compared to lower nanocellulose addition in the UF resin, probably owing to cellulose particle aggregation. In both cases, the presence of nanocellulose led to lower HCHO emissions from the prepared boards. This study demonstrates that it is possible to disperse (nano)cellulose derived by the simple sonication of a parent microcrystalline cellulose with no subsequent modification into the UF matrix to be further used for the enhancement of particle boards. Therefore, time-demanding and costly processes of cellulose treatment, either by chemical modification with organosilane agents for improved compatibility or by altering its liquid state to solid (freeze-drying, spray-drying, etc.) for improved dispersion, can be avoided. Finally, we demonstrate that ultrasonication in nanocellulose production can serve as an alternative method for the fabrication of cellulose nanoparticles with promising properties and strong polymer-reinforcing potential.

## Data Availability

Data are contained within the article or Supplementary Materials.

## Supplementary Materials

The following supporting information can be downloaded at: https://www.mdpi.com/article/10.3390/molecules30142911/s1. Figure S1: UF resins’ properties before (UF reference) and after (NCS) nanocellulose addition in different concentrations. Figure S2: SEM images of the cross-sections of particle boards prepared with the use of the UF reference resin and of nanocellulose-reinforced UF_NCS resin. Figure S3: DSC curves of particle boards with UF reference resin and UF_NCS nanocellulose-containing resin, during (a) 1st heating at 10 °C/min, (b) cooling at 10 °C/min, and (c) 2nd heating at 10 °C/min. References [36,37] are cited in the supplementary materials.

## References

[1] Antunes A., Paiva N., Ferra J., Martins J., Carvalho L., Barros-Timmons A., Magalhães F.D. (2018). Highly Flexible Glycol-Urea-Formaldehyde Resins. Eur. Polym. J..

[2] Kawalerczyk J., Walkiewicz J., Dziurka D., Mirski R., Brózdowski J. (2022). APTES-Modified Nanocellulose as the Formaldehyde Scavenger for UF Adhesive-Bonded Particleboard and Strawboard. Polymers.

[3] Wibowo E.S., Park B.-D. (2021). Crystalline Lamellar Structure of Thermosetting Urea-Formaldehyde Resins at a Low Molar Ratio. Macromolecules.

[4] Myers G.E. (1984). How Mole Ratio of Uf Resin Affects Formaldehyde Emission and Other Properties: A Literature Critique. For. Products J..

[5] Pizzi A., Lipschitz L., Valenzuela J. (1994). Theory and Practice of the Preparation of Low Formaldehyde Emission Uf Adhesives. Holzforschung.

[6] Wibowo E.S., Park B.-D. (2021). Direct Measurement of Surface Adhesion between Thin Films of Nanocellulose and Urea–Formaldehyde Resin Adhesives. Cellulose.

[7] Dorieh A., Selakjani P.P., Shahavi M.H., Pizzi A., Movahed S.G., Pour M.F., Aghaei R. (2022). International Journal of Adhesion and Adhesives Recent Developments in the Performance of Micro/Nanoparticle-Modified Urea-Formaldehyde Resins Used as Wood-Based Composite Binders: A Review. Int. J. Adhes. Adhes..

[8] Papadopoulos L., Malletzidou L., Patsiaoura D., Magaziotis A., Psochia E., Terzopoulou Z., Chrissafis K., Markessini C., Papadopoulou E., Bikiaris D.N. (2021). Synthesis and Characterization of Unsaturated Succinic Acid Biobased Polyester Resins. Appl. Sci..

[9] Liu C., Wang C., Tang J., Zhang J., Shang Q., Hu Y., Wang H., Wu Q., Zhou Y., Lei W. (2018). High-Performance Biobased Unsaturated Polyester Nanocomposites with Very Low Loadings of Graphene. Polymers.

[10] Pizzi A., Papadopoulos A.N., Policardi F. (2020). Wood Composites and Their Polymer Binders. Polymers.

[11] Toriz G., Arvidsson R., Westin M., Gatenholm P. (2003). Novel Cellulose Ester–Poly(Furfuryl Alcohol)–Flax Fiber Biocomposites. J. Appl. Polym. Sci..

[12] El Oudiani A., Msahli S., Sakli F. (2017). In-Depth Study of Agave Fiber Structure Using Fourier Transform Infrared Spectroscopy. Carbohydr. Polym..

[13] Turki A., El Oudiani A., Msahli S., Sakli F. (2022). Infrared Spectra for Alfa Fibers Treated with Thymol. J. Glycobiol..

[14] Yang X., Han F., Xu C., Jiang S., Huang L., Liu L., Xia Z. (2017). Effects of Preparation Methods on the Morphology and Properties of Nanocellulose (NC) Extracted from Corn Husk. Ind. Crop. Prod..

[15] Margellou A.G., Psochia E.A., Torofias S.A., Pappa C.P., Triantafyllidis K.S. (2024). Isolation of Highly Crystalline Cellulose via Combined Pretreatment/Fractionation and Extraction Procedures within a Biorefinery Concept. AcsSustain. Resour. Manag..

[16] Silva A.C.Q., Silvestre A.J.D., Vilela C., Freire C.S.R. (2022). Cellulose and Protein Nanofibrils: Singular Biobased Nanostructures for the Design of Sustainable Advanced Materials. Front. Bioeng. Biotechnol..

[17] Psochia E.A., Delliere P., Sanchez R., Triantafyllidis K.S., Guigo N. (2024). Sustainable Alliance between Nanocellulose and Biobased Poly Ur Uryl Alcohol. Ind. Crops Prod..

[18] Inamuddin, Thomas S., Mishra R.K., Asiri A.M. (2019). Sustainable Polymer Composites and Nanocomposites.

[19] Veigel S., Rathke J., Weigl M., Gindl-Altmutter W. (2012). Particle Board and Oriented Strand Board Prepared with Nanocellulose-Reinforced Adhesive. J. Ofnanomaterials.

[20] Mahrdt E., Pinkl S., Schmidberger C., Van Herwijnen H.W.G., Veigel S., Gindl-Altmutter W. (2015). Effect of Addition of Microfibrillated Cellulose to Urea-Formaldehyde on Selected Adhesive Characteristics and Distribution in Particle Board. Cellulose.

[21] Karagiannidis E., Markessini C., Athanassiadou E. (2020). Micro-Fibrillated Cellulose in Adhesive Systems for the Production of Wood-Based Panels. Molecules.

[22] Kojima Y., Kato N., Ota K., Kobori H., Suzuki S., Aoki K., Ito H. (2018). Cellulose Nanofiber as Complete Natural Binder for Particleboard. For. Prod. J..

[23] Iglesias M.C., McMichael P.S., Asafu-Adjaye O., Via B.K., Peresin M.S. (2021). Interfacial Interactions between Urea Formaldehyde and Cellulose Nanofibrils (CNFs) of Varying Chemical Composition and Their Impact on Particle Board (PB) Manufacture. Cellulose.

[24] Hansted F.A.S., Hansted A.L.S., Padilla E.R.D., Caraschi J.C., Goveia D., De Campos C.I. (2019). The Use of Nanocellulose in the Production of Medium Density Particleboard Panels and the Modification of Its Physical Properties. BioResources.

[25] Júnior R.R.M., Cardoso G.V., Ferreira E.S., Costa H.L. (2020). Surface characterization, mechanical and abrasion resistance of nanocellulose-reinforced wood panels. Surf. Topogr. Metrol. Prop..

[26] Amoroso L., Muratore G., Ortenzi M.A., Gazzotti S., Limbo S., Piergiovanni L. (2020). Fast Production of Cellulose Nanocrystals by Hydrolytic-Oxidative Microwave-Assisted Treatment. Polymers.

[27] Zinge C., Kandasubramanian B. (2020). Nanocellulose Based Biodegradable Polymers. Eur. Polym. J..

[28] Wang Q., Yao Q., Liu J., Sun J., Zhu Q., Chen H. (2019). Processing Nanocellulose to Bulk Materials: A Review. Cellulose.

[29] Indran S., Raj R.E., Sreenivasan V.S. (2014). Characterization of New Natural Cellulosic Fiber from Cissus Quadrangularis Root. Carbohydr. Polym..

[30] Yu H., Qin Z., Liang B., Liu N., Zhou Z., Chen L. (2013). Facile Extraction of Thermally Stable Cellulose Nanocrystals with a High Yield of 93% through Hydrochloric Acid Hydrolysis under Hydrothermal Conditions. J. Mater. Chem. A.

[31] Papadopoulou E., Kountouras S., Nikolaidou Z., Chrissafis K., Michailof C., Kalogiannis K., Lappas A.A. (2016). Urea-Formaldehyde (UF) Resins Prepared by Means of the Aqueous Phase of the Catalytic Pyrolysis of European Beech Wood. COST Action FP1105. Holzforschung.

[32] Moslemi A., Koohi M.Z., Behzad T., Pizzi A. (2020). Addition of Cellulose Nanofibers Extracted from Rice Straw to Urea Formaldehyde Resin; Effect on the Adhesive Characteristics and Medium Density Fiberboard Properties. Int. J. Adhes. Adhes..

[33] Zhang H., She Y., Song S.P., Pu J.W. (2013). Modified Nanocrystalline Cellulose Used for Improving Formaldehyde Emission and Bonding Strength of Urea Formaldehyde Resin Adhesive. Key Eng. Mater..

[34] Dunky M. (1998). Urea-Formaldehyde Adhesive Resins for Wood. Int. J. Adhes. Adhesives..

[35] Moutousidis D., Karidi K., Athanassiadou E., Stylianou E., Giannakis N., Koutinas A. (2023). Sustainable Chemistry for the Environment Reinforcement of Urea Formaldehyde Resins with Pectins Derived from Orange Peel Residues for the Production of Wood-Based Panels. Sustain. Chem. Environ..

[36] Kawalerczyk J., Dziurka D., Dukarska D., Woźniak M., Walkiewicz J., Mirski R. (2024). The effect of urea-formaldehyde adhesive modification with diisocyanate-functionalized nanocellulose on the properties of particleboard. Int. J. Adhes. Adhes..

[37] Kong X., Wei Z., Xia S., Jia B., Gan L., Han S. (2023). The characterizations of nanofluid type urea formaldehyde resins. Int. J. Adhes. Adhes..

