# Supplementary material for: Reinforcing Urea–Formaldehyde Resins with Low-Cost, Mechanically Derived Nanocellulose: A Sustainable Approach
Source: Molecules. 2025 Jul 10;30(14):2911. doi: 10.3390/molecules30142911 (PMC12300287; doi:10.3390/molecules30142911)
Supplement: Supplementary file 1 [file molecules-30-02911-s001.zip › molecules-3733347-supplementary.pdf]

Reinforcing Urea–Formaldehyde Resins with Low-Cost, Mechanically Derived Nanocellulose: A Sustainable Approach

Eleni A. Psochia [1,*], Emmanouil Karagiannidis [2], Eleftheria Athanasiadou[2] and Konstantinos S. Triantafyllidis [3,4,*]

[1] Department of Chemistry, Aristotle University of Thessaloniki, 54214 Thessaloniki, Greece; epsochia@gmail.com
[2] CHIMAR HELLAS S.A.,15 km National Road, Thessaloniki–Polygyros, 57001 Thessaloniki, Greece; Manos.Karag@ari.gr, eathan@ari.gr
[3] Chemistry Department, King Fahd University of Petroleum and Minerals, Dhahran 31261, Saudi Arabia
[4] Interdisciplinary Research Center for Refining and Advanced Chemicals, King Fahd University of Petroleum & Minerals, Dhahran 31261, Saudi Arabia; k.triantafyllidis@kfupm.edu.sa
*Correspondence: epsochia@gmail.com; k.triantafyllidis@kfupm.edu.sa

SUPPORTING INFORMATION

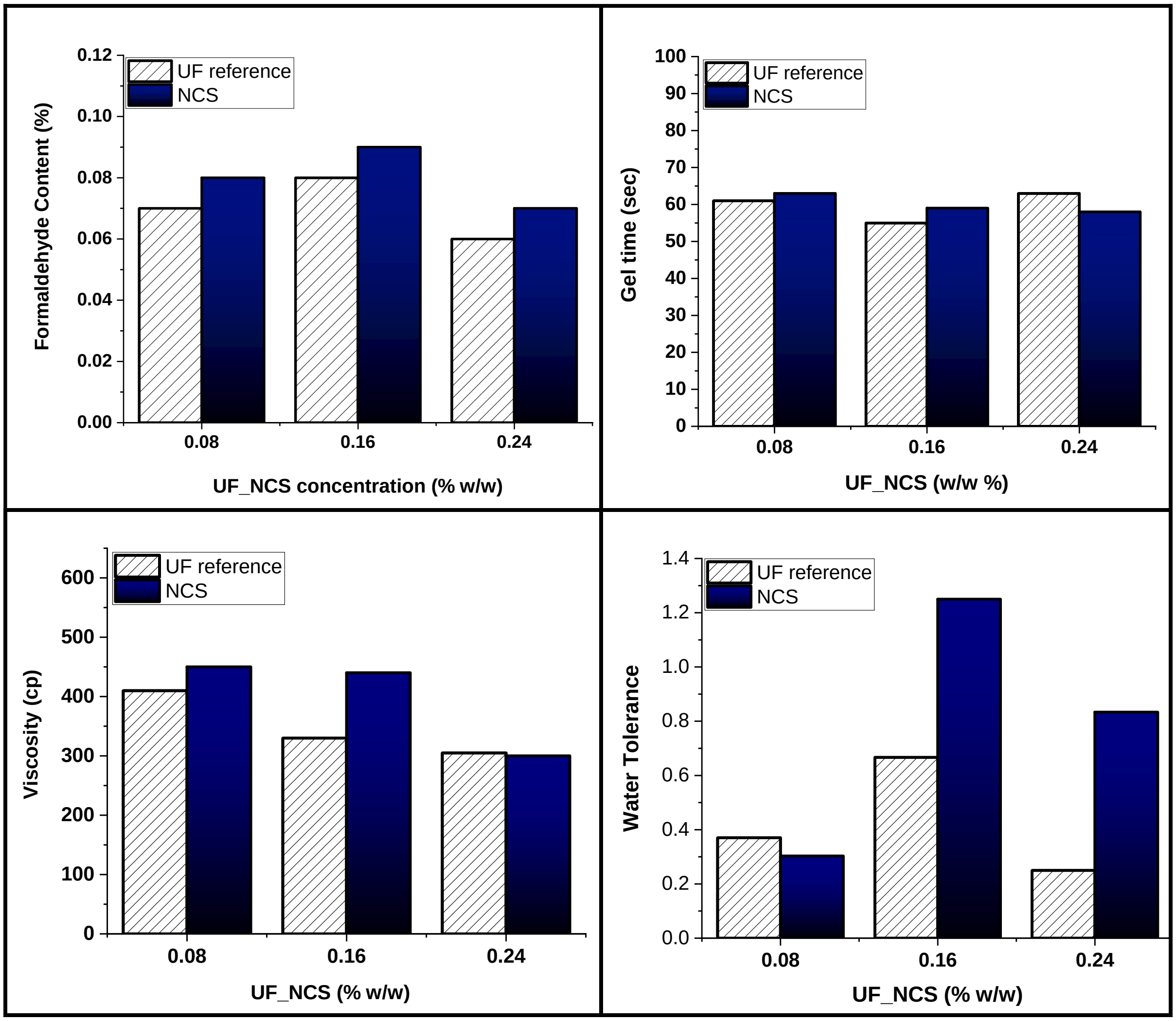

Figure S1. UF Resins' properties before (UF reference) and after (NCS) nanocellulose addition in different concentrations.

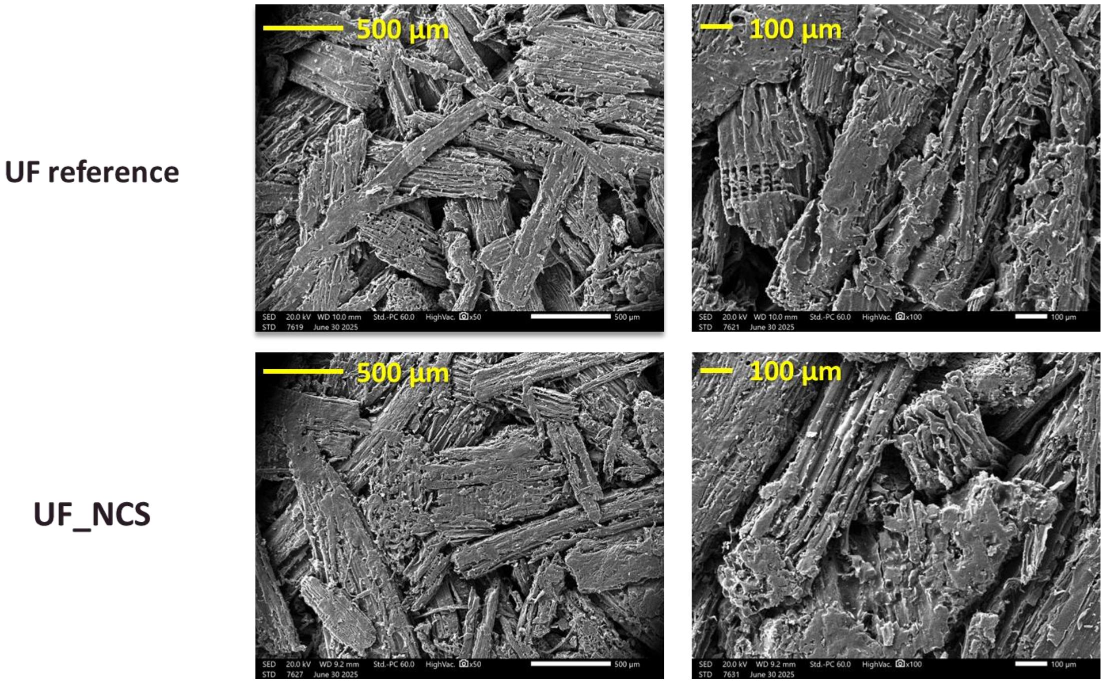

Figure S2. SEM images of the cross sections of particle boards prepared by the use of UF reference resin and of nanocellulose reinforced UF_NCS resin.

No significant morphological differences can be observed between the two samples. Furthermore, as expected, no distinct nanocellulose fibers or particles can be distinguished, due to very low nanocellulose loading levels in the adhesive resin (⩽ 0.24 wt % on a dry-resin basis), considering also that the UF resin (or the nanocomposite UF resin) itself constitutes ~10 wt% of the final board, resulting in effective nanocellulose content in the particle board of < 0.03 wt%.

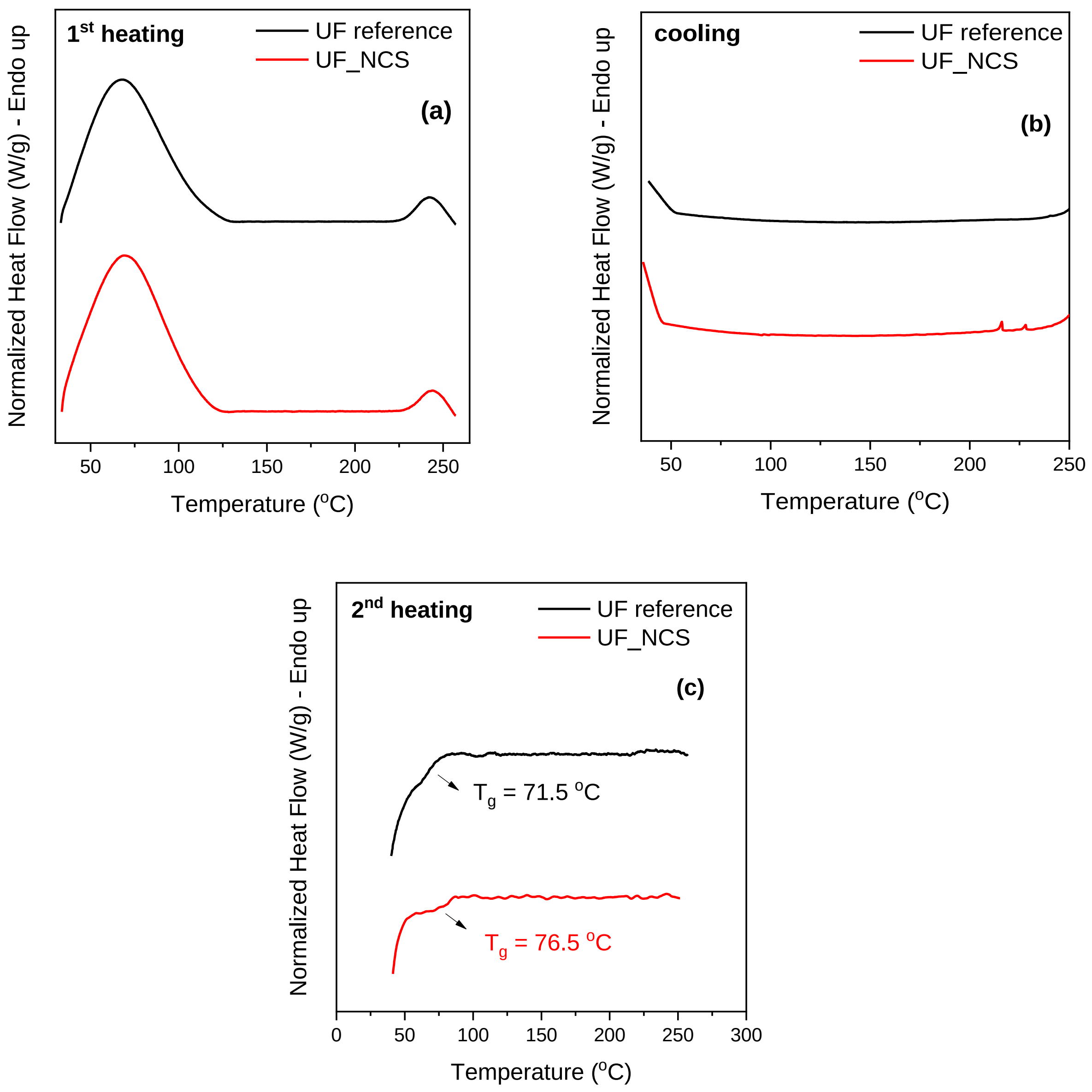

Figure S3. DSC curves of particle boards with UF reference resin and UF_NCS nanocellulose containing resin, during **(a)** 1st heating at 10 °C/min, **(b)** cooling at 10 °C/min and **(c)** 2nd heating at 10 °C/min.

In the 1st heating ramp no significant differences between the particle boards with UF reference resin and UF_NCS nanocomposite resin can be observed (Figure 2a). An endothermic peak at ~70 °C is observed for both particle boards, owing probably to further curing of the bulk UF resin. A smaller endothermic peak for both samples was also observed at around 230-250 °C. This could be associated with thermal degradation of unreacted UF components, decomposition of the cured UF network as well as decomposition of the carbohydrates of the lignocellulosic wood chips included in the particle board. During the cooling ramp no crystallization phenomenon for both samples was observed (Figure 2b). Finally, from the second heating ramp, a slightly difference in the glass transition temperature (T_g) could

be observed. The board containing the UF reference resin exhibited a T_g of around 71.5 °C, while after nanocomposite resin containing board showed a T_g shift to slightly higher temperature, ca. to 76.5 °C. This indicates that the presence of nanocellulose hinders the polymer chain mobility due to the hydrogen bonding formation between its surface hydroxyl groups and the resins' hydroxyl or amine groups, as discussed in the main text and supported by ^{13}C-NMR analysis. The effect of nanocellulose on increasing the T_g of the UF resins has also been shown in other studies in literature [1], [2].

References

[1] J. Kawalerczyk, D. Dziurka, D. Dukarska, M. Woźniak, J. Walkiewicz, and R. Mirski, "The effect of urea-formaldehyde adhesive modification with diisocyanate-functionalized nanocellulose on the properties of particleboard," *International Journal of Adhesion and Adhesives*, vol. 135, no. October, 2024, doi: 10.1016/j.ijadhadh.2024.103850.

[2] X. Kong, Z. Wei, S. Xia, B. Jia, L. Gan, and S. Han, "The characterizations of nanofluid type urea formaldehyde resins," *International Journal of Adhesion and Adhesives*, vol. 126, no. July, p. 103451, 2023, doi: 10.1016/j.ijadhadh.2023.103451.
